# Tutorials in population neuroimaging: Using epidemiology in neuroimaging research

**DOI:** 10.3389/fnimg.2022.934514

**Published:** 2022-08-05

**Authors:** Sara Godina, Mini E. Jacob, Mary Ganguli

**Affiliations:** ^1^Department of Epidemiology, University of Pittsburgh, Pittsburgh, PA, United States; ^2^Department of Psychiatry and Behavioral Sciences, University of Washington, Seattle, WA, United States; ^3^Department of Neurology, University of Pittsburgh, Pittsburgh, PA, United States; ^4^Department of Psychiatry, University of Pittsburgh, Pittsburgh, PA, United States

**Keywords:** neuroimaging, epidemiology, neuroscience, research design, methods

## Abstract

Epidemiology is the foundation of all public health research and practice. Epidemiology confers many important uses for the advancement of neuroimaging research. Epidemiology serves as a framework to organize pieces of data and guide critical thinking in the research process from the early stages of study design to the end goal of reaching appropriate inferences. Epidemiology accounts for the profound heterogeneity in populations, thoroughly describes study samples, and identifies consequential threats to study validity. Finally, epidemiology is a discovery tool that can lead researchers to uncover new risk factors, disease states, and subpopulations. The neuroimaging investigator with a grasp of the principles of epidemiology is in a unique position to undertake valid clinical epidemiology and etiological research.

## Introduction

The researcher who wants to study a neurological condition or disease using neuroimaging methods has an appreciation of the key characteristics of the condition, specifically, causes, pathophysiology, clinical manifestations, and prognosis. Although this foundational knowledge is a critical asset for the successful neuroimaging investigator, it is not sufficient to design a research study that is valid and reliable. The Five Ws (who, what, when, where, why) are taught in journalism as the necessary pieces of information for any story to be complete. An epidemiological framework will challenge neuroimaging researchers to ask similar questions for a more complete research picture: participant (who), health exposure or outcome (what), timing (when), 12pt place (where), and risk factors (why). It is imperative for neuroimaging researchers to critically think about who the individuals are in their study: where have they been before getting in a scanner? What exposures have they encountered, when, and for how long? How are these specific individuals different from the target population, and how will that impact inference and validity of conclusions? Even with the best possible neuroimaging methodology and a thorough understanding of the pathophysiology of the disease, the study's results may not be reliable if the source of data and sampling strategies lead to a sample that is not representative. For example, compared to a community hospital, a specialized clinic may attract primarily high-income participants with high socioeconomic status. Sampling is not only important in geographic terms, but also temporally, in instances when disease trends change over time due to changes in the environment, diet availability, etc. The discipline of epidemiology serves to address such challenges. The thoughtful neuroimaging investigator, armed with an understanding of the principles of epidemiology, can combine cutting-edge neuroimaging methodologies with state-of-the-art study design and is in a particularly advantageous position to advance scientific knowledge through neuroimaging research on vital clinical questions.

### What is epidemiology?

Classically, epidemiology is defined as “(1) the study of the distribution and determinants of health-related states and events in populations; and (2) the application of this study to the prevention and control of health problems” (Jacob and Ganguli, 2016). The field of epidemiology is frequently described as a “bridge” between basic biological science and population prevention policies. As we know it, the field of epidemiology originated in a milestone study of an outbreak of cholera by physician John Snow in 1850s in England. By discovering that cholera was being spread throughout London *via* consumption of contaminated water from the (now infamous) Broad Street pump, Snow transformed how the pathogenesis of cholera was understood. Since Snow's breakthrough, epidemiologic and public health research has led to revolutionizing measures to improve the health of populations, ranging from eradication of smallpox through vaccination to transforming HIV-AIDS from a terminal disease to a manageable chronic disease through antiretroviral treatment.

Past the investigation of communicable diseases, applying the same principles, epidemiology has expanded to understand chronic diseases at the population level. Advances in technology have led to the development of subspecialty fields, such as genetic epidemiology and geospatial epidemiology. However, the translational process of taking lab discoveries and turning them into quantifiable health benefits in human populations is complicated. Basic and clinical neuroscience research has yielded pivotal advancements in our understanding of the structure and function of the human brain, it has become obvious that researchers must look beyond small convenience samples of patients and toward larger representative samples to explore the pathogenesis of diseases and conditions of the central nervous system. This is the underlying motivation for the field of “population neuroscience,” which aims to merge the knowledge base and skill sets of epidemiologists with those of neuroscientists (Paus, 2010; Falk et al., 2013).

### Population neuroscience

Population neuroscience seeks to leverage interdisciplinary expertise and limitations of both epidemiology and neuroscience. Incorporating neuroscience measures into well-characterized epidemiologic cohorts allows for the examination of mechanisms that underlie the associations between exposures and outcomes. The application of epidemiologic methods to neuroscience variables will further allow for consideration of mediators and moderators along the hypothesized pathways and improved generalizability due to population-based sampling methods. Six concrete goals to advance such an integrative and collaborative framework between neuroscience and epidemiology have been outlined by Falk et al. (2013) (Table 1) and describe how team-based science can begin to link analyses from the neuronal level up to the population level.

**Table 1:** Goals to advance population neuroscience (Falk et al., 2013).

1. Integrate brain imaging into existing representative (sub)samples
2. Development of methods to scale up neuroimaging studies to larger and more representative samples with methods allowing for cross-study, cross-age, and cross-culture comparisons
3. Use strategic sampling when recruiting for stand-alone neuroimaging studies
4. Explore moderators of brain-behavior links and neural predictors of relevant outcomes
5. Changing of the cultures in neuroscience and population research
6. Emphasis on development and ecological interactional models

## Epidemiology serves as a guiding research framework

### Study designs

A clear and concise question should be the start of any research proposal, which in turn determines an appropriate study design. An investigator might employ the use of the “patient/population, intervention, comparison, outcome, timing, setting (PICOTS)” mnemonic as a starting framework to define the necessary components of a clinical or healthcare-related question: PICOTS. After a well-defined research question has been formulated, an investigator is able to select an appropriate study design, and further, appropriate inferences can be drawn from results, based on design. Thus, before any participants are even recruited for a study and/or data collected, a thoughtful investigator will be able to delineate the strengths and weaknesses of their chosen study design.

Epidemiologists use an assortment of different study designs to estimate associations among social, environmental, or biologic predictors and health outcomes. Generally, these study designs can be classified in two ways: observational or experimental (Table 2). As the name would suggest, in observational studies, participants are observed without researcher intervention, and data are collected on the variables needed to test the research question. In contrast, the researcher intervenes in experimental studies to try and influence a health outcome. Here, we briefly discuss the most common study designs, the corresponding effect measures, and the conclusions of each design.

**Table 2:** Classification of epidemiologic studies.

Observational
Descriptive
Analytic
Ecologic
Cross-Sectional
Case–Control
Cohort
Experimental
Randomized controlled trials
Community trials
Field trials

#### Observational studies

##### Descriptive studies

A descriptive study describes an outcome in relation to its magnitude and distribution by person, place, and time, but does not formally estimate any exposure/outcome associations. For example, the tabulation of the mortality rate due to stroke in a country over several decades is used to describe the mortality trend over time. More formal analytic studies are necessary to properly evaluate the factors associated with a trend and potentially determine its causes. Purely descriptive studies are rare; a descriptive analysis is often the preliminary stage of an epidemiologic study that eventually tests associations.

##### Cross-sectional studies

In cross-sectional studies, both the predictor and outcome are measured in a sample of the population at a single time point, akin to a “snapshot.” Cross-sectional studies are frequently used because they are time, money, and resource efficient, and typically low burden to participants (and researchers). The low burden is especially important for participants of advanced age, who might not be in great health, and might be unable or unwilling to make it to repeated research visits. Time efficiency is important to aging researchers studying diseases of aging that exhibit steep rates of decline in health. Cross-sectional studies are further beneficial for researchers in the aging field because we can include many individuals across the lifespan in the same study. However, since exposure and outcome are measured at the same time, we cannot establish temporality in cross-sectional studies, or assert that exposure is causally related to outcome in any way. Furthermore, any average pulled from cross-sectional data could be misrepresentative of the true data, as an assumption in cross-sectional designs is that changes from one age group to another mirror changes from one individual to another (and we know the aging process is highly heterogeneous); cross-sectional and longitudinal data might not agree with each other. However, given all of these limitations, consistent exposure/outcome associations that have been observed in cross-sectional studies are usually the first evidence of a “signal” of a “true” relationship before more advanced study designs are employed.

##### Case–control studies

In case–control studies, a group of individuals with identified disease (cases) is compared to a group of individuals without the disease (controls). Cases are typically recruited based on referrals in a clinical setting or by media advertisement, from a broadly defined population (e.g., residents of a given city). The same broadly defined population criteria are used to select controls, so controls can be as similar as possible to the cases with the exception of having the disease. Appropriate control selection is a critical aspect of case–control studies; if systematic differences between cases and controls exist in characteristics other than the disease itself, the “true” association between the exposure and disease will be obscured. The association may be completely missed or if there is no association, a spurious association may be observed. Similarly, it is important to collect data from both cases and controls in a similar fashion. For example, it would be inappropriate if the exposure data was indirectly obtained from family members of the cases but directly from the case. For both cases and controls, exposure data are typically captured through self-report, clinical examination, or laboratory tests. An odds ratio is the effect measure used when estimating the association between an exposure and outcome in a case–control study; the odds ratio is a reasonable approximation of the relative risk for rare diseases. Case–control study designs are ideal for an investigator to use when studying a rare disease that might have too low a prevalence to be cost-effectively detected by traditional random sampling methods.

##### Cohort studies

Cohort studies prospectively follow a representative group of individuals over time (longitudinally). Typically, a group without disease or outcome of interest is followed over time to detect incident cases of disease as they arise. At both study entry and follow-up assessments, exposure status is measured, and the entire cohort is assessed for disease development. As new cases are detected, it is possible to calculate an incidence rate, and relative risk is used to measure associations between first exposure and later disease.

Although cohort studies are an ideal study design for investigating predictors of diseases and health outcomes, they are burdensome with respect to money and time. It is challenging and labor-intensive to recruit and retain a random sample of participants over a follow-up of many years. Participant drop-out impacts the internal validity of a cohort study, as attrition potentially introduces bias. Individuals who do not drop-out or are not lost to follow-up in a high-burden longitudinal study are likely to be significantly different from other participants that do drop-out and the greater target population.

#### Experimental studies

Experimental studies involve testing the effects of an intervention in a sample of individuals. Disease or health outcomes are compared between those that received the intervention under study and those that did not. Ethical considerations must be at the forefront of a researcher's mind when designing and implementing an experimental study. Randomized controlled trials (RCTs) are the most common experimental study design.

##### Randomized controlled trials

Randomized controlled trials randomly allocate whether participants receive an intervention or not. Theoretically, randomization helps to ensure that at study entry the intervention and control groups are comparable, e.g., there is no bias in selection for intervention.

### Epidemiologic inference

#### Sample size

Whether actively designing a new study or reading a research manuscript, it is imperative to understand the ways in which results are easily influenced based on sample size. With an increasing number of participants in a study, there is an increasing likelihood that the sample is representative of the source population. In contrast, smaller samples are more vulnerable to the influence of variability throughout the sampling process, as human populations are largely heterogeneous. Given the difficulty and expense of recruiting a large and random sample, sample size calculation is a critical factor to consider in study design. Such calculations will yield the minimum number of participants needed to test a specific hypothesis or research question. When calculating sample size, the following values are considered.

#### P-Value

The ***p***-value is the probability that an observed association is due to chance, assuming the null hypothesis is true and no other biases (information, selection, or confounding) are present in the study. Random error is always possible, as even when individuals are identified ***via*** random sampling methods, the ultimate study sample can still be different or show associations that are not found in the parent population. Conventionally, an alpha of 0.05 is utilized in studies to constrain the probability of this error. Such a threshold minimizes the chance of rejecting a “true” null hypothesis, e.g., incorrectly interpreting a chance finding as genuine (also known as alpha or type 1 error). The ***p***-values are sensitive to the characteristics of the study sample and might be low for many reasons. The ***p***-value changes with confounding, selection, or information bias, as the line that defines a “significant” threshold in the distribution shifts with the introduction of bias. The ***p***-values are also impacted by sample size, as sample size increases, the ***p***-value decreases. Finally, a very low ***p***-value might be evidence of the null hypothesis not being true, and that there is a “true” association underlying the data; your data are highly unlikely with a true null hypothesis, these numbers would rarely occur by chance alone. Historical over reliance and misinterpretation of ***p***-values have been a recent hot topic in the scientific community, with some scientists going so far as to call for the abandonment of statistical significance altogether (Amrhein et al., 2019).

#### Normal distribution and confidence limits

Here, we assume that readers are familiar with the concepts of normal distribution, central tendency, and dispersion. An underlying assumption of many formal statistical tests commonly used to estimate differences between groups is that variables of interest are normally distributed in the target population of interest. However, even if this is not the case, many statistical tests will be sufficiently robust, given a large enough sample size. Conventionally, a sample size of 30 or greater, with no fewer than five individuals per compared subgroup is considered appropriate (Pett, 1997; Salkind, 2004).

The 95% confidence limits are corollaries of the ***p***-value threshold of 0.05 described above. In the absence of bias (information, selection, or confounding), if we were to repeat the experiment 100 times, the estimated 95% confidence interval would include the true value 95 times out of 100. However, we do know whether this interval contains the “true” estimate or not. To determine whether or not there is a “signal” in the confidence interval, we can interpret from a precision point, and how close estimates range together. However, again, this assumes no bias as estimates can be very precise and/or similar but still biased. The more narrow confidence intervals are, the more precise the corresponding estimates will be. In general, the greater the sample size, the more narrow the confidence interval will be.

#### Power

Power is the probability of correctly detecting a difference between groups in a sample, given said difference exists in the parent population. Conventionally, power is set in a range of 80–95% to minimize the possibility of beta or type 2 error, e.g., the probability of accepting a false null hypothesis or missing a true difference. The relationship between a study's power, type 1 and type 2 errors, all within the context of the conclusions drawn vs. “true reality,” is shown in Table 3.

**Table 3:** Power, Type I, and Type II error.

		**“Truth”**	
		**Null hypothesis false**	**Null hypothesis true**
Conclusion	Reject null hypothesis	True Positive (Power, 1-β)	False Positive (Type 1 error, α)
	Accept null hypothesis	False Negative Type 2 error (β)	True Negative

#### Effect size

The estimate of the effect size captures the magnitude of the difference between study groups. Effect size is a necessary component of sample size calculations; more power (larger sample size) is needed to detect smaller differences in effect size. In such calculations, expected effect sizes are typically estimated based on prior research findings and/or clinical relevance.

## Epidemiology accounts for the profound heterogeneity in populations

### Sampling

Once a thoughtful research question has been defined, an appropriate study is designed, and consideration of how power and sample size will impact any conclusions, a neuroimaging researcher, critiquing their study through the lens of an epidemiologist, might then turn their attention to a thorough discussion of the participants who make it into their study, and again, how these specific participants impact appropriate inferences.

#### Random sampling

Employing random sampling techniques ensure that each individual in the target population of interest has an equal chance of selection. Researchers must carefully consider the impacts of drawing their samples from certain populations. For example, it is perfectly reasonable to estimate the prevalence of stroke in a clinic sample or dementia in a nursing home sample, given the researcher takes into account that estimates will likely be substantially higher in such settings, compared to the general public. Furthermore, since individuals in clinics and nursing homes themselves are not randomly drawn from all individuals with stroke or dementia in the community, there are inherent selection biases and consequential limitations on appropriate inferences that can be generalized to the target population of interest. Observational or experimental studies that employ convenience samples of patients or volunteers with strict eligibility criteria and burdensome study procedures are similarly vulnerable to such selection factors and generalizability of findings.

#### Validity

The word “generalizability” is frequently used as a default criticism or limitation, since no study is fully representative of (i.e., generalizable to) all populations. Internal validity can be compromised if the variables under investigation and selection factors are associated with themselves. Sometimes these associations are unavoidable, such as an age effect where older adults with an age-related disease or health outcome are less likely to participate in research compared to younger adults with the same disease or outcome. Other times, the investigator imposes selection factors through study design or eligibility, e.g., if criteria for participation in an Alzheimer's disease (AD) study exclude individuals with stroke, any association between stroke and AD will be unable to be studied.

For a research study to have external validity, appropriate population sampling is needed so the sample is representative of the larger target population that it purports to be drawn from. Replication of results is a cornerstone of the scientific process; however, it is expected that not every replication study will yield similar results. For example, we would not expect that results from a study of traumatic brain injury (TBI) outcomes in teachers would be generalizable to similar research in military veterans, given inherent differences in exposures and TBI rates in the two groups. However, given appropriate sampling, it is reasonable to expect results from a sample of teachers would be true of the larger population of teachers where the study sample was drawn from and ultimately to the target population of all teachers. Furthermore, if similar exposure/outcome associations were found in both populations (teachers and veterans), there is a greater likelihood that the findings are reflective of a true relationship (Kukull and Ganguli, 2012).

#### Representative samples

To reiterate, the ultimate purpose of drawing a representative sample is so a researcher can appropriately extrapolate that the results from the study sample are also “true” in the larger population from which it was drawn. Ideally, the average value of a characteristic (e.g., brain volume) measured in the study sample would also be the average value of that characteristic found in the larger population. Furthermore, this holds true for predictors and exposures of interest. For example, the proportion of individuals in the sample population with a certain attribute (e.g., hypertension) should be similar to the underlying proportion in the larger population.

### Threats to study validity

#### Bias

Bias is a consequence of systematic error found within the design and conduct of a study, wherein observed and “true” results differ. Typically, studies are vulnerable to bias in the chosen method of participant recruitment (selection) and/or in the measurement of exposure or outcome. We describe two general categories of bias: selection and information bias.

##### Selection bias

Selection bias occurs if systematic differences exist between individuals in the population selected for participation in a study, compared to those in the population who are not selected. For example, there is a larger proportion of individuals with AD who carry the APOE^*^4 genotype if the study recruits from a research clinic, compared with surveillance population estimates within the same area (Tsuang et al., 1996). Subsequently, it was found that compared to population estimates, individuals with AD recruited from a clinic were more likely to exhibit characteristics associated with the allele: they tended to be younger, with earlier Alzheimer's onset, and in a more advanced disease state. Thus, this underlying selection bias found in the individuals recruited from a clinic sample yielded a biased estimate of the relative risk and was overestimated, compared to the risk in the larger population.

Prevalence or length bias is a selection bias that occurs because prevalent cases of a disease or health outcome are found in individuals who have survived to a certain point in time. Similar to the selection bias discussed earlier, prevalence bias can over or under estimate associations between predictors and outcomes. Many case–control studies from the 1990s consistently showed a protective association between smoking status and odds of AD (Kukull, 2001). Taking prevalence bias into consideration, it was later discovered that smokers who developed AD died earlier when compared to non-smokers who developed AD, due to other adverse health outcomes associated with smoking. The apparent protective association was induced because of an inflated proportion of controls who smoked and a reduced proportion of cases who smoked. This phenomenon is also known as “competing risks” where a predictor of interest (here, smoking) is associated with multiple outcomes (here, AD and death), and the occurrence of one outcome (death) prevents the researcher from observing the other outcome (AD).

Attrition bias is another important selection bias to consider, especially in longitudinal study designs. Study participants that are lost to follow-up over time are likely to be different when compared to participants that stay in the study until completion. For example, participants that are lost to follow-up in a study due to death are likely more critically ill, compared to individuals who survive long enough to complete the study. Ignoring the impact of this selection and assuming individuals were lost to follow up at random would bias results and effect estimates. Realistically, attrition is unavoidable in most studies, and there are methodologies available to capture and address the impact of attrition bias on a study's results. Inverse probability weighting is the most common method to address the problem of attrition in cohort studies. Inverse probability of attrition weighting utilizes logistic regression methods to model the likelihood of participant study dropout, so the researcher can determine the extent to which dropout might have impacted the observed results.

##### Information bias

Information bias occurs when there are inaccuracies in the quantification and/or classification of exposure and outcome. Case–control studies are vulnerable to a type of information bias termed recall bias, as cases are more likely to report previous exposures compared to controls. Several previous case–control studies of AD and head trauma showed an association (Mortimer et al., 1991) that could not be replicated in prospective study designs when exposure status is captured prior to dementia onset (Chandra et al., 1989). Observer bias occurs when the person measuring the disease or health outcome has knowledge of the subject's exposure status, (inaccurately) influencing the measurement. Blinding investigators to exposure status minimizes the likelihood of observer bias.

##### Confounding

**Figure 1:**
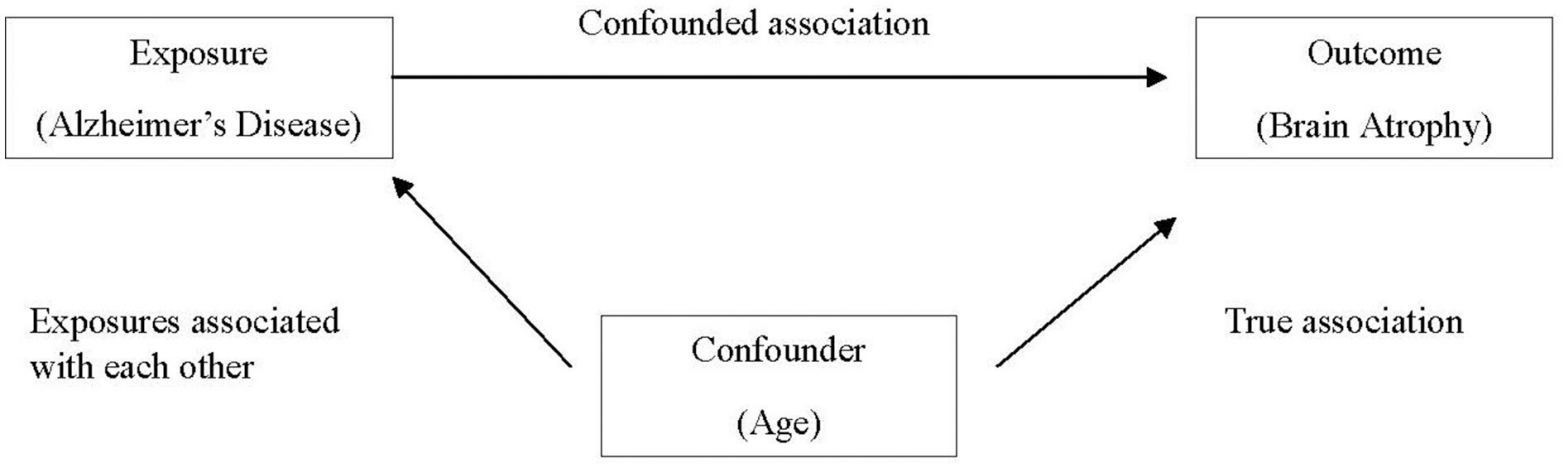
Age as a confounder in the associationbetween Alzheimer's disease and brain atrophy.

Confounding occurs when a certain exposure “A” is associated with both the exposure of interest “B” and the disease or health outcome of interest “C,” and its effect has not been appropriately separated out. Erroneously, a researcher might conclude that exposure and outcome of interest are associated, when in fact the relationship is spurious and induced due to the third variable. An apparent association between AD and brain atrophy might be confounded by age, as older individuals are more likely to have brain atrophy and are also more likely to have AD (Franke et al., 2010), as demonstrated in Figure 1.

There are several methods to control confounding in the study design stage, including randomization, matching, and restriction. As discussed earlier, experimental studies frequently employ random allocation of intervention (i.e., randomization) in an effort to balance potential confounding factors between intervention and control groups. In case–control studies, matching cases and controls on confounding factors (frequently age, sex, race, etc.) are commonly employed. Restriction utilizes exclusion criteria to eliminate potential confounders in the selection of participants. When analyzing data, confounders can be controlled by including them as covariates in statistical models and/or running separate analyses for individuals with and without the potential confounder (stratification).

Even after using the above strategies to mitigate the influence of confounding, there is likely to be a degree of persistent confounding, as it is not feasible for every confounder to be identified and/or measured—we call this residual (unmeasured) confounding.

##### Effect modification

In contrast to confounding, when the magnitude of the effect between exposure and outcome of interest differs depending on the level of a third variable, effect modification (interaction) is present. If effect modification is present and a researcher (erroneously) computes an overall estimate, the estimate will again be distorted, as when confounding is present. Effect modification is best examined by stratifying estimates for each level of the third variable. However, it is important for the researcher to keep in mind that the choice to stratify results in a consequential loss of power.

##### Mediation

For a third variable to be considered a mediator, it must exist on the causal pathway between exposure and outcome. Mediation analysis methods are available to disentangle both direct and indirect effects of a third variable in the exposure-outcome association.

#### Strategies to mitigate bias

So far, we have discussed how both systematic and random errors are inherent in scientific research. Weaknesses in methodology and/or execution of a study that can affect validity are considered a systematic error. As opposed to random error, there are tools to quantitatively measure and avoid systematic errors. Quantitative bias analyses (QBAs) are used to estimate direction, magnitude, and uncertainty resulting from systematic error (Lash et al., 2014).

## Epidemiology is a discovery tool

At the most basic level, epidemiologic research questions surround testing an association between a specified exposure and the outcome of interest in human populations. Asking such questions in well-designed studies with large, generalizable samples and finding consistent results lead to estimates of an individual's chance and risk of the outcome of interest. By employing population studies, we have more precise estimates (and confidence) to extrapolate down to the individual level. Then, based on consistent results gleaned from epidemiologic studies, researchers can use the information on individual morbid risk to evaluate the efficacy of treatment options and preventive strategies. But, without valid computation of individual morbid risk (and risk factors), we would be unable to effectively judge treatments and prevention level interventions. Again, if we see consistent results across well-designed epidemiologic studies (starting with descriptive cross-sectional work, moving into cohort and case–control, and final the “gold standard” of randomized controlled trials), we have the confidence to speak to the necessary community intervention and policies to treat and prevent such an outcome.

### Causal inference

#### Establishing causality

The process of establishing whether an observed exposure/outcome association is reflective of a “true” underlying cause-and-effect relationship is termed “causal inference.” It is complicated to attempt to establish causation; theoretically, we could only truly determine causality if we were to examine the exact same group of participants with and without exposure (simultaneously) and observe any disease or health outcome. This counterfactual framework is impossible in the real world; however, experimental RCTs are our best approximation of such a scenario. Of course, it is not possible to randomize every exposure, e.g., we cannot randomize participants to smoke or not smoke, or to experience or not experience stroke. In addition to advanced statistical methods, epidemiologists use several other tools to help distinguish between association and causation, including directed acyclic graphs (causal diagrams) and Bradford-Hill criteria (Table 4). Although the use of any of these tools can help a researcher's confidence in taking their results from association to causation, triangulation of the complete body of evidence from both animal and human studies is typically necessary to establish causality.

**Table 4:** Bradford-Hill criteria.

1. Strength of association
2. Consistency of data
3. Specificity
4. Temporality
5. Dose-Response
6. Biological plausibility
7. Coherence
8. Experimental evidence
9. Analogy

### Completing the clinical picture

Diseases are classically first observed and described in clinical settings. However, due to the aforementioned selection factors that influence the type of individuals that make it to such settings, individuals seen in clinical settings are likely to be atypical when compared to those with the same disease in the larger population. In the early 1900s, Alois Alzheimer detailed the pathology and symptomology of a singular case study of presenile dementia in a 51-year-old woman. Consequentially, the disease was considered to be an uncommon disease in middle-aged individuals (Cipriani et al., 2011). It would not be until the 1960s that Alzheimer's disease would be established as a relatively common disease among older adults, after a population-based neuropathologic study by Roth et al. (1966).

### Delineating new syndromes

Parkinsonism and Guillain–Barré syndrome were initially identified in clinical settings and were discussed as case studies or series in the scientific literature. Subsequently, the development of case definitions and delineation of subtypes came out of epidemiologic studies. For example, an epidemiologic study of a large sample of individuals with Guillain–Barre syndrome in China determined that acute motor axonal neuropathy as an important subtype (Mckhann et al., 1993). A large population-based study established psychological and behavioral disturbances as common features of dementia, when they had previously been disregarded in clinical research (Lyketsos et al., 2000). It is imperative to employ a clinical epidemiology approach, so these distinct subtypes of disease can be identified, based on differing clinical presentation, response to therapeutics, and pathogenesis. Epidemiologic methods have also promoted the identification and prioritization of phenomena, which do not always directly present to a clinician, e.g., subclinical cardiovascular disease (Chaves et al., 2004). Epidemiology helped to link metabolic syndrome (Reaven, 1997) and frailty syndrome (Fried et al., 2001), two syndromes previously thought to be disparate phenomena.

#### Cohort effects

Cohort effects are variations over time, in one (or more) factors, among groups of people defined by a shared event such as birth year, or dates of specific exposure. Any given population contains numerous subcohorts with differing exposure and outcome rates. Thus, although the larger population might appear heterogeneous, smaller subcohorts might show more homogeneity that was previously masked in the larger cohort. For example, an association between age and cognitive impairment may be reflective of a cohort effect and not an age effect. Individuals born in earlier cohorts grew up during the Depression Era when many boys stopped their schooling in their pre-teens to work in the coal mines. Subsequent poor cognition in their late life could result from either their lack of secondary education and/or environmental exposures from the coal mines, in contrast to later generations, and is not simply a function of “age.” In addition to cohort and age effects, period effects are due to events that happened at a specific time, e.g., a nuclear radiation exposure, or the development of a new therapeutic class of drugs.

When assessing trends over time, other factors to consider include changes in screening and/or diagnostic criteria and changes in the age of the population. If any of these factors are present, they can inappropriately induce changes in incidence and/or prevalence estimates, which are not indicative of a true trend due to, e.g., introduction of a new therapeutic treatment of disease.

## Putting it all together: Relevance to the neuroimaging researcher

A recent review of over one-thousand brain magnetic resonance imaging (MRI) papers over two decades (1990–2012) found the majority of highly cited experimental MRI studies average sample sizes of <50 participants (Szucs and Ioannidis, 2020). Furthermore, the same review evaluated more recent practices (2017–2018) and found only a minority of highly cited neuroimaging research report power calculations and specification of effect sizes (Szucs and Ioannidis, 2020). Given such small sample sizes and consequential low power, it is likely that some highly cited neuroimaging research has a high likelihood of type 1 and type 2 errors. These compounding issues ranging from study design to sample populations make it problematic to draw appropriate inferences from the data, let alone to the target population. Thoughtful use of epidemiologic methods and principles can help the field of neuroscience overcome such common limitations.

Thus far, we discussed at some length how the use of epidemiologic principles can inform neuroimaging research. It is important to close with a brief discussion of a few common “misuses” of epidemiology. These are common practices reflective of a misunderstanding of the principles of epidemiology we described. First, the use of “epidemiologic” to denote a specific study design instead of a global framework for understanding and examining health outcomes at the population level. Second, using cross-sectional data to make directional inferences. Third, generalizing results to a larger target population based on data from biased (non-representative) samples. Fourth, making the incorrect assumption that an observed exposure/outcome relationship is causal (i.e., an observed association is a signal). Fifth, not taking timing and duration of exposure into account when describing recommendations and future interventions, based on observational data. Finally, failing to consider biological plausibility and/or underlying mechanisms when reporting associations.

Here, we summarized the main strengths of incorporating epidemiology into neuroimaging research, along with motivating examples from the literature. Using an epidemiologic framework, with the knowledge of intentional sampling and selection of appropriate study design, identification of threats to study validity, and strategies on how to mitigate bias, the thoughtful neuroimaging researcher will be better equipped to incorporate population methods into their own work.

## Author contributions

All authors contributed to the production of this manuscript and have approved the final version.

## Funding

SG was supported by the NIH award (T32AG055381) and the K. Leroy Irvis Fellowship at the Graduate School of Public Health, University of Pittsburgh.

## Conflict of interest

The authors declare that the research was conducted in the absence of any commercial or financial relationships that could be construed as a potential conflict of interest.

## Publisher's note

All claims expressed in this article are solely those of the authors and do not necessarily represent those of their affiliated organizations, or those of the publisher, the editors and the reviewers. Any product that may be evaluated in this article, or claim that may be made by its manufacturer, is not guaranteed or endorsed by the publisher.

## References

[B1] Amrhein V. Greenland S. Mcshane B. (2019). Scientists rise up against statistical significance. Nature 567, 305–307. 10.1038/d41586-019-00857-9 30894741

[B2] Chandra V. Kokmen E. Schoenberg B. S. Beard C. M. (1989). Head trauma with loss of consciousness as a risk factor for Alzheimer's disease. Neurology 39, 1576–1578. 10.1212/WNL.39.12.1576 2586772

[B3] Chaves P. H. Kuller L. H. O'leary D. H. Manolio T. A. Newman A. B. (2004). Subclinical cardiovascular disease in older adults: insights from the cardiovascular health study. Am. J. Geriatr. Cardiol. 13, 137–151. 10.1111/j.1076-7460.2004.02120.x 15133417

[B4] Cipriani G. Dolciotti C. Picchi L. Bonuccelli U. (2011). Alzheimer and his disease: a brief history. Neurol. Sci. 32, 275–279. 10.1007/s10072-010-0454-7 21153601

[B5] Falk E. B. Hyde L. W. Mitchell C. Faul J. Gonzalez R. Heitzeg M. M.. (2013). What is a representative brain? Neuroscience meets population science. Proc. Natl. Acad. Sci. U.S.A. 110, 17615–17622. 10.1073/pnas.1310134110 24151336 PMC3816464

[B6] Franke K. Ziegler G. Klöppel S. Gaser C. (2010). Estimating the age of healthy subjects from T1-weighted MRI scans using kernel methods: exploring the influence of various parameters. NeuroImage 50, 883–892. 10.1016/j.neuroimage.2010.01.005 20070949

[B7] Fried L. P. Tangen C. M. Walston J. Newman A. B. Hirsch C. Gottdiener J.. (2001). Frailty in older adults: evidence for a phenotype. J. Gerontol. A Biol. Sci. Med. Sci. 56, M146–M156. 10.1093/gerona/56.3.M146 11253156

[B8] Jacob M. E. Ganguli M. (2016). Epidemiology for the clinical neurologist. Handb. Clin. Neurol. 138, 3–16. 10.1016/B978-0-12-802973-2.00001-X 27637949

[B9] Kukull W. A. (2001). The association between smoking and Alzheimer's disease: effects of study design and bias. Biol. Psychiatry 49, 194–199. 10.1016/S0006-3223(00)01077-5 11230870

[B10] Kukull W. A. Ganguli M. (2012). Generalizability: the trees, the forest, and the low-hanging fruit. Neurology 78, 1886–1891. 10.1212/WNL.0b013e318258f812 22665145 PMC3369519

[B11] Lash T. L. Fox M. P. Maclehose R. F. Maldonado G. Mccandless L. C. Greenland S. (2014). Good practices for quantitative bias analysis. Int. J. Epidemiol. 43, 1969–1985. 10.1093/ije/dyu149 25080530

[B12] Lyketsos C. G. Steinberg M. Tschanz J. T. Norton M. C. Steffens D. C. Breitner J. C. (2000). Mental and behavioral disturbances in dementia: findings from the cache county study on memory in aging. Am. J. Psychiatry 157, 708–714. 10.1176/appi.ajp.157.5.708 10784462

[B13] Mckhann G. M. Cornblath D. R. Griffin J. W. Ho T. W. Li C. Y. Jiang Z.. (1993). Acute motor axonal neuropathy: a frequent cause of acute flaccid paralysis in China. Ann. Neurol. 33, 333–342. 10.1002/ana.410330402 8489203

[B14] Mortimer J. A. Van Duijn C. M. Chandra V. Fratiglioni L. Graves A. B. Heyman A.. (1991). Head trauma as a risk factor for Alzheimer's disease: a collaborative re-analysis of case-control studies. EURODEM risk factors research group. Int. J. Epidemiol. 20 (Suppl. 2), S28–35. 10.1093/ije/20.Supplement_2.S28 1833351

[B15] Paus T. (2010). Population neuroscience: why and how. Hum. Brain Mapp. 31, 891–903. 10.1002/hbm.21069 20496380 PMC6871127

[B16] Pett M. A. (1997). Nonparametric Statistics for Health Care Research: Statistics for Small Samples and Unusual Distributions. Thousand Oaks, CA: Sage.

[B17] Reaven G. M. (1997). Banting lecture 1988. Role of insulin resistance in human disease 1988. Nutrition 13, 65. 10.1016/0899-9007(97)90878-9 9058458

[B18] Roth M. Tomlinson B. E. Blessed G. (1966). Correlation between scores for dementia and counts of 'senile plaques' in cerebral grey matter of elderly subjects. Nature 209, 109–110. 10.1038/209109a0 5927229

[B19] Salkind N. J. (2004). Statistics for People Who (Think They) Hate Statistics. Thousand Oaks, CA: Sage.

[B20] Szucs D. Ioannidis J. P. A. (2020). Sample size evolution in neuroimaging research: an evaluation of highly-cited studies (1990–2012) and of latest practices (2017–2018) in high-impact journals. NeuroImage 221, 117164. 10.1016/j.neuroimage.2020.117164 32679253

[B21] Tsuang D. Kukull W. Sheppard L. Barnhart R. L. Peskind E. Edland S. D.. (1996). Impact of sample selection on APOE epsilon 4 allele frequency: a comparison of two Alzheimer's disease samples. J. Am. Geriatr. Soc. 44, 704–707. 10.1111/j.1532-5415.1996.tb01836.x 8642164

